# JS-K, a nitric oxide pro-drug, regulates growth and apoptosis through the ubiquitin-proteasome pathway in prostate cancer cells

**DOI:** 10.1186/s12885-017-3351-0

**Published:** 2017-05-26

**Authors:** Guobin Tan, Mingning Qiu, Lieqian Chen, Sai Zhang, Longzhi Ke, Jianjun Liu

**Affiliations:** Laboratory of Urology, Guangdong Medical College, Zhanjiang, Guangdong 524001 China

**Keywords:** Prostate cancer, JS-K, Ubiquitin E3 ligase, Apoptosis, Proliferation

## Abstract

**Background:**

In view of the fact that JS-K might regulate ubiquitin E3 ligase and that ubiquitin E3 ligase plays an important role in the mechanism of CRPC formation, the goal was to investigate the probable mechanism by which JS-K regulates prostate cancer cells.

**Methods:**

Proliferation inhibition by JS-K on prostate cancer cells was examined usingCCK-8 assays. Caspase 3/7 activity assays and flow cytometry were performed to examine whether JS-K induced apoptosis in prostate cancer cells. Western blotting and co-immunoprecipitation analyses investigated JS-K’s effects on the associated apoptosis mechanism. Real time-PCR and Western blotting were performed to assess JS-K’s effect on transcription of specific AR target genes. Western blotting was also performed to detect Siah2 and AR protein concentrations and co-immunoprecipitation to detect interactions of Siah2 and AR, NCoR1 and AR, and p300 and AR.

**Results:**

JS-K inhibited proliferation and induced apoptosis in prostate cancer cells. JS-K increased p53 and Mdm2 concentrations and regulated the caspase cascade reaction-associated protein concentrations. JS-K inhibited transcription of AR target genes and down-regulated PSA protein concentrations. JS-K inhibited Siah2 interactions and also inhibited the ubiquitination of AR. With further investigation, JS-K was found to stabilize AR and NCoR1 interactions and diminish AR and p300 interactions.

**Conclusions:**

The present results suggested that JS-K might have been able to inhibit proliferation and induce apoptosis via regulation of the ubiquitin-proteasome degradation pathway, which represented a promising platform for the development of new compounds for PCa treatments.

**Electronic supplementary material:**

The online version of this article (doi:10.1186/s12885-017-3351-0) contains supplementary material, which is available to authorized users.

## Background

Nitric oxide (NO), which was found in 1987 to be a physiological constituent, and in the following years, found to be synthesized in vivo, work as a signal molecule, toxicant, and antioxidant with a broad spectrum of actions among physiological and pathological processes [1]. NO shows pro and anti-cancer abilities depending on the cell type, conditions, NO source, concentration, and NO release rate [2, 3]. As it appears to have a crucial role in tumor biology, controlling tumor growth, migration, invasion, and angiogenesis, modulating NO-signaling might be a promising strategy in cancer treatments [4–7].

Chemical agents with stabilize NO release have been developed as NO’s limitations, such aqueous solubility and instability in the presence of various oxidants, have become better understood. One such effective NO pro-drug is JS-K(O2-(2,4-dinitrophenyl)-1-[(4-ethoxycarbonyl)piperazin-1-yl]diazen-1-ium-1,2-diolate), a new nitric oxide donor that belongs to the diazeniumdiolate family of compounds. It has been designed to release NO within a cell in a sustained and controlled manner during its reaction with glutathione-S-transferase (GST), which is often overexpressed in cancer cells [8]. Recently, increasing evidence has suggested that JS-K regulates tumor occurrence and development of tumor, such as leukemia, prostate cancer, hepatoma, multiple myeloma, and lung cancer in vitro and in vivo [9–12]. However, the underlying mechanism by which JS-K influences prostate cancer cells remains unclear.

Prostate cancer (PCa) is the most commonly diagnosed neoplasm in elderly men and the second greatest cause of cancer-related deaths in the United States [13]. Androgen ablation therapies, such as orchiectomy, systemic administration of LHRH analog/blocker or anti-androgen, are the primary treatments for advanced PCa. Although such endocrine therapies have achieved significant clinical responses, patients with advanced PCa eventually relapse with a more aggressive PCa form, which is defined as castration-resistant PCa (CRPC). Intensive studies of CRPC pathogenesis have shown that PCa recurrence is implicated in resumption of AR-dependent transcriptional activity. Dramatically, Qi et al. have found that the ubiquitin ligase E3 Siah2playsan important role in AR action regulation in CRPC. Interestingly, Siah2 is markedly overexpressed in human CRPC and found to work as a regulator for the inactive AR chromatin complexes well as to mediate degradation, thus resulting in activation of AR-regulated genes involved in cell proliferation, cell motility, and lipid metabolism. One focus throughout their study was that Siah2-dependent removal of NCoR1-bound AR allows p300-bound AR binding to androgen receptor elements (AREs) of AR target genes [14].

The ubiquitin-proteasome pathway works in multiple steps. First, ubiquitin is activated from its precursor by addition to the ubiquitin-activating enzyme (E1); second, the activated ubiquitin is transferred to the ubiquitin-conjugating enzyme (E2); third, E2 interacts with ubiquitin-protein ligase (E3) and transfers ubiquitin to the target protein and ubiquitin; and finally, selective tagging and degradation of specific intracellular proteins are allowed according to the type of ubiquitin modification on protein substrates [15–17]. Although gene transcription and ubiquitin-mediated proteolysis are two processes that seemingly have nothing in common, a growing body of evidence has indicated that the ubiquitin-proteasome pathway is intimately involved in regulating gene transcription [18]. Qi et al. have suggested that Siah2 is a crucial mediator for reconditioning chromatin regions that govern AR-dependent transcription through degradation of inactive AR-NCoR1 complexes on promoter regions of AREs [14]. Meanwhile, NCoR1 is a known AR co-repressor [19], which promotes interactions between active AR-p300complexes and AREs. As is known, this process promotes CRPC formation [14].

Interestingly, Chen et al. [20] have shown that the abnormal ubiquitination process is found during tumor formation. Strikingly, a research article published in *Oncogene* has shown thatMdm2 is an ubiquitin ligase E3 that auto-ubiquitylates itself and also ubiquitylates p53, resulting in degradation of both proteins. Furthermore, JS-K inhibits Mdm2-mediated p53 ubiquitylation, leading to p53 accumulation in Tert-immortalized, human retinal pigment, epithelial (RPE) cells [21]. Thus, it is possible that JS-K inhibition on PCa might have been achieved by regulating the ubiquitin-proteasome pathway. In view of the fact that JS-K regulates the stability and activity of ubiquitin ligase E3 Siah2 and that Siah2 plays such an important role in CRPC progression, the goal of this study was to investigate the probable mechanism by which JS-K inhibits Siah2-regulated AR responsive genes that contribute to CRPC.

## Methods

### Cell culture

Human prostate cancer cell lines LNCaP was obtained from Shanghai Institute of Biochemistry and Cell Biology (SIBCB, Shanghai, China) and C4-2 was obtained from American Type Cell Culture (ATCC, USA), all of which were AR-positive. Prostate cancer cells were routinely grown in RPMI-1640 medium GIBCO, Grand Island, NY, USA, supplemented with 10% fetal bovine serum (FBS, GIBCO), 100 U/ml penicillin, and 100 U/ml streptomycin at 37 °C under an atmosphere of 5% CO_2_ in humidified air.

### Cell proliferation assay

Proliferation of LNCaP and C4-2 cells was evaluated by Cell Counting Kit-8 (CCK-8, Dojindo, Japan) assay according to the manufacturer’s instructions. Briefly, Cells (1 × 10^3^/well) were plated in 96-well plates (Corning Incorporated; Corning, NY, USA) for 3 days, and treated by JS-K (5 μM) for 12, 24 and 48 h. 10μLCCK-8reagentwas added to the culture medium in each well. After incubating at 37 °C for 3 h, absorbance at 450 nm of each well was measured with a microplate reader (BioTek Instruments, Inc., USA). Each experiment was repeated three times, and the data represent the mean of all measurements.

### Real time quantitative PCR (RT-PCR)

Total RNA was isolated using the total RNA kit (Omega Bio-tek, Inc., Guangzhou, China) and reversely transcribed to cDNAs with a TaqMan miRNA Reverse Transcription Kit (TaKaRa, Dalian, Liaoning, China). The mRNA levels of *Siah2*, *NKX3.1*, *PSA*, *PMEPA1*, and *SLC45A3*were quantified by real-time quantitative PCR performed with SYBR Premix Ex Taq II (TaKaRa; Dalian, Liaoning, China). PCR was carried out with a two-step qRT-PCR with specific primers for *GAPDH* (as internal control) at 95 °C for 30s, followed by 40 cycles of amplification at95°C for 5 s and 56 °C for 30s. All results were representative of three independent assays, and the levels of mRNAs were expressed as 2^-ΔΔCT^. The designed specific primers were listed in Table 1.

**Table 1:** Sequences for target gene primer for RT-PCR

Gene		Primer sequence 5′-3’	Tm (°C)
siah2	F:	GCCCACAAGAGCATTACCAC	59.80
	R:	GTTTCTCCAGCACCAGCAT	57.60
NKX3.1	F:	GCCAAGAACCTCAAGCTCAC	59.80
	R:	TTCTCCAAGTCTCCCAGCTC	59.80
PMEPA1	F:	CTCCACCACACACACATCG	59.70
	R:	CGCCTTCCTCTCACTCCTCT	61.90
SLC45A3	F:	GAGCCGAGACGAAGCAGTT	59.70
	R:	GCCAAAGGTTAGCAGGTTGA	57.80
PSA	F:	TCCTCACAGCTGCCCACT	60.58
	R:	ATATCGTAGAGCGGGTGTGG	59.98

### Caspase-3/7 activity assay

For Caspase-3/7 activity assays, LNCaP and C4-2cells were treated by JS-K in time-dependent manner and Caspase-Glo 3/7 assay was performed in 96-well plates. Then, an equal volume of Caspase-Glo 3/7 reagent was added into each well, and the cells were incubated for 30 min at room temperature in the dark. The luminescence was measured by a luminometer (Berthold Sirius L, Germany).

### Apoptosis analysis

FITC Annexin V Apoptosis Detection Kit I (BD Biosciences, USA) was used to access the apoptosis of PCa cells induced by JS-K according to the manufacturer protocols.

### Western blotting analysis

Western blotting was conducted using standard procedures, the membrane was incubated with anti-PARP (Cell Signaling Technology, USA), anti-p53 (Santa Cruz Biotechnology, Europe), anti-Bcl-2 (Cell Signaling Technology, USA), anti-Bax (Cell Signaling Technology, USA), anti-Caspase-9 (Cell Signaling Technology, USA), anti-Caspase-3 (Cell Signaling Technology, USA), anti-AR (Santa Cruz Biotechnology, Europe), anti-Siah2 (Santa Cruz Biotechnology, Europe), anti-NCoR1 (Santa Cruz Biotechnology, Europe), anti-p300 (Santa Cruz Biotechnology, Europe), Mdm2 (Santa Cruz Biotechnology, Europe), anti-Ub (Cell Signaling Technology, USA), anti-GADPH (Abcam, Cambridge, MA, USA). And then the membrane strip were probed with a secondary antibody (1:10,000, Pure Earth Biotechology Co. Ltd.), GADPH was used as a loading control.

### Co-immunoprecipitation

Cells were washed with PBS prior to cell lysis in 1 ml of IP lysis buffer [20 mM Tris pH 7.5, 150 mM NaCl, 1% Triton X-100, 2.5 mM sodium pyrophosphate, 1 mM EDTA, 1% Na_3_VO_4_, 0.5 μg/mL leupeptin, 1 mM phenylmethanesulfonyl fluoride (PMSF)], and Cell lysates were cleared by centrifuging at 14,000×g for 10 min at 4 °C. After the supernatant was incubated overnight at 4 °C with suitable dilutions of the primary antibody, Protein A/G Agarose (Beyotime Institute of Biotechnology, Haimen, China) was added, and incubated for additional 4 h at 4 °C. Washed precipitated proteins were analyzed by Western blot.

### Statistical analysis

Each experiment was done at least twice and at least one duplicate. The results were presented as mean ± standard deviation(SD). All statistical analyses were performed using SPSS 17.0. Differences between treatments were assessed using Fisher’s Least Significant Difference test [LSD (L)]. Significant difference was inferred for *P* < 0.05 and extremely significant difference *P* < 0.01 and *P* < 0.001.

## Results

### JS-K regulated PCa cell proliferation and apoptosis

**Fig. 1:**
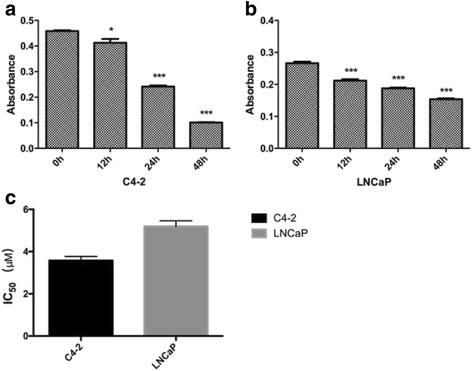
The inhibitory effect of JS-K on proliferation of C4-2 cells (**a**) and LNCaP (**b**). C4-2 and LNCaP cells were treated with indicated concentration of JS-K (5 μM) for three periods (12, 24 and 48 h). The viability of cells was measured by CCK-8 assay. (**c**) The IC_50_ of JS-K for LNCaP and C4-2 cell lines were tested, while the cells were treated with JS-K (0, 1, 2, 5, 10 and 20 μM) for 48 hours. Each assay was performed in triplicate. Results are mean ± SD of three different experiments. Single asterisks (*) indicate a significant difference (*P* < 0.05) and triple asterisks (***) indicates an extremely significant difference (*P* < 0.001)

First, the inhibitory effects of proliferation by JS-K onC4-2 and LNCaP cells were investigated. JS-K inhibited growth of C4-2 and LNCaP cells in a time-dependent manner (Fig. 1a and b, respectively). As JS-K treatment at 5 μM(IC_50_, Fig. 1c, Additional file 1: IC50 results) showed a significant effect on C4-2 and LNCaP cell proliferation, JS-K at 5 μM was chosen as the representative dose for treatment in vitro in subsequent studies.

**Fig. 2:**
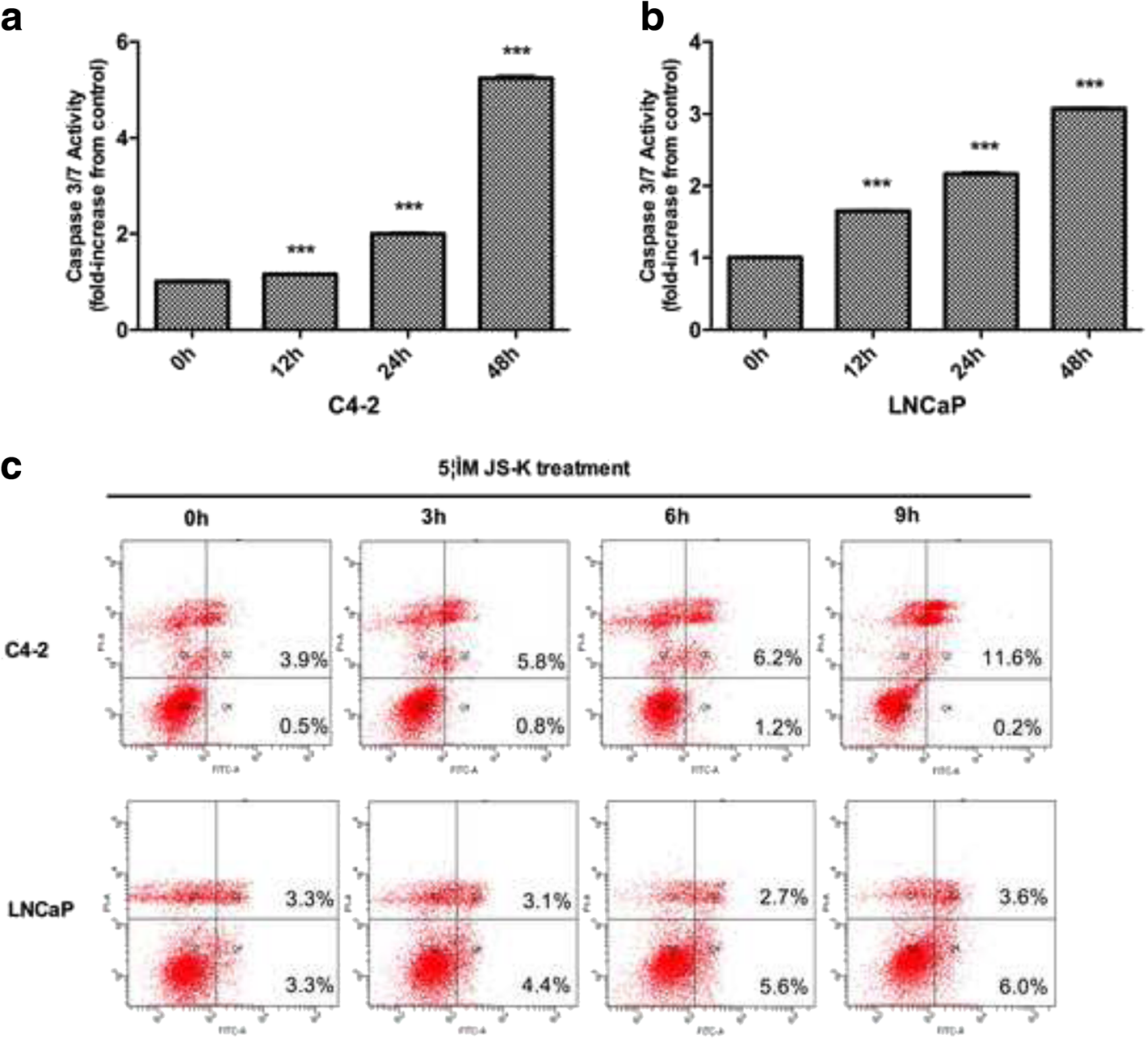
Caspase 3/7 activity assay. C4-2 cells (**a**) and LNCaP (**b**) were treated with indicated concentration of JS-K (5 μM) for three periods (12, 24 and 48 h). The apoptosis of cells was detected by Caspase 3/7 activity assay (Promega) and each assay was performed in triplicate. **c** Apoptosis induced by JS-K in PCa cells treated with indicated concentration of JS-K (5 μM) for three periods (3, 6 and 9 h) was analyzed by flow cytometry with the Annexin V staining method. Untreated cells were analyzed as control. Data indicated that JS-K could induce apoptosis in PCa cells in a time-dependent manner. Results are mean ± SD of three different experiments. Triple asterisks (***) indicates an extremely significant difference (*P* < 0.001)

As significant inhibitory effects of JS-K on LNCaP and C4-2 cells was observed, caspase-Glo 3/7 assays were performed to investigate whether JS-K induced apoptosis in PCa cells. JS-K treatment at 5 μM for 12, 24, or 48 h resulted in increased caspase 3/7 activity (*p* < 0.001, Fig. 2a and b). Similar effects were observed in LNCaP cells. In addition, flow cytometry was used to investigate the effect of JS-K on PCa cell apoptosis (Fig. 2c), which detected an increased apoptotic ratio in these cells in a time-dependent manner.

### JS-K appeared to regulate apoptosis-associated mechanisms via the ubiquitin proteasome pathway

**Fig. 3:**
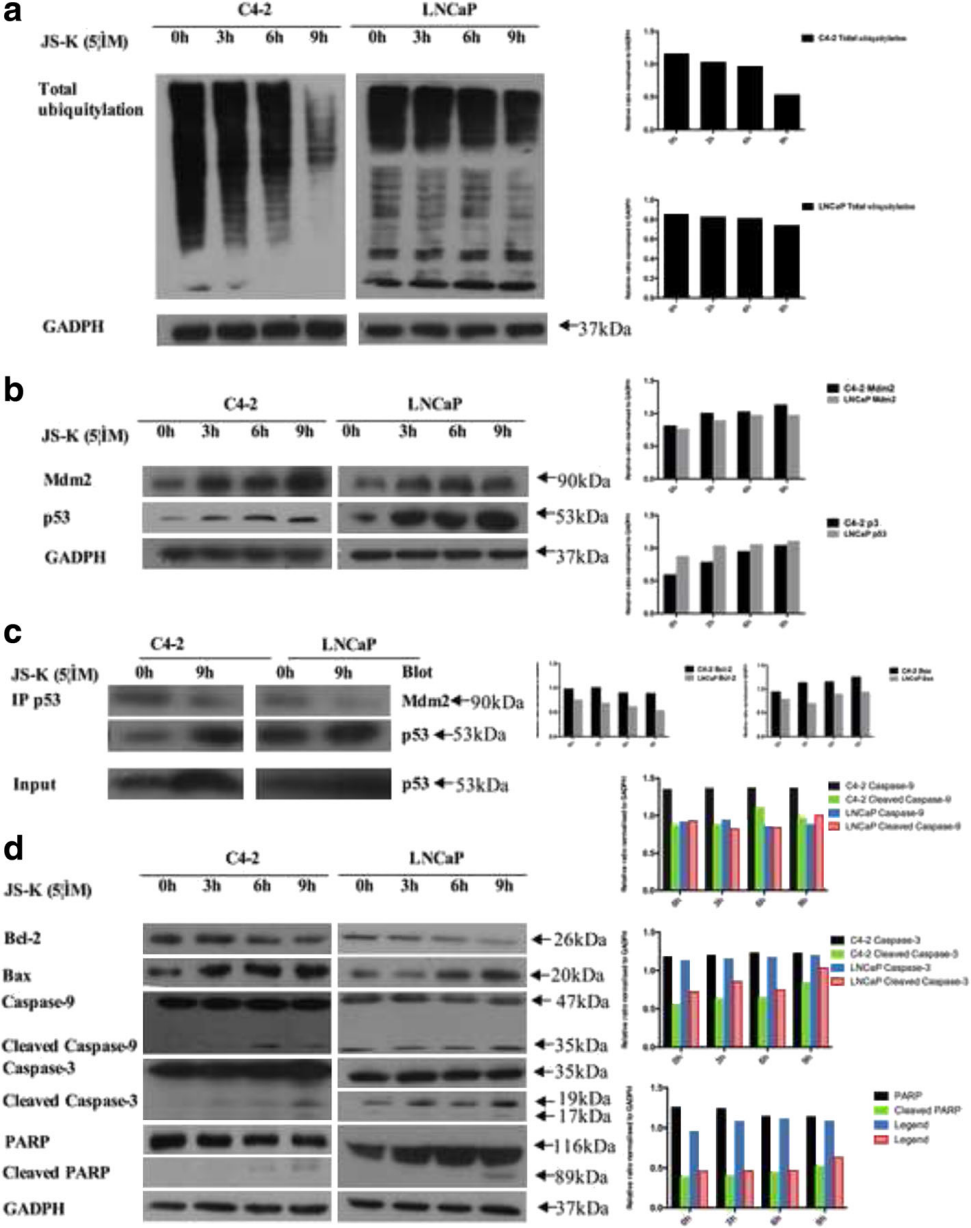
C4-2 and LNCaP cells were incubated for three periods (3, 6 and 9 h) with 5 μM JS-K. **a** Total ubiquitination protein was diminished by JS-K; **b** JS-K increased p53, Mdm2 protein levels; **c** JS-K inhibited the combination of p53 and Mdm2 that were detected by Co-IP; All the IP were used in the input and at least three different experiments were performed; **d** apoptosis relative proteins (PARP, Bcl-2, Bax, Caspase-9 and Caspase-3) were detected by western blotting. GADPH was set as the loading control

Wild-type p53 is a tumor suppressor protein and significant regulator in cell growth that is considered to be stable and accumulate in DNA-damaged cells. Increasing evidence has indicated that the ubiquitin E3 ligase Mdm2 inhibits p53 activity by ubiquitin proteolysis [22]. A valuable JS-K study has implicated that JS-K induces DNA damage and thus increases p53 expression concentrations, which activates apoptosis involved in the Bcl-2, Bax, and caspase cascade reactions [23]. Because Mdm2 mediatesp53 proteolysis and JS-K inhibits Mdm2 activity [21, 22], JS-K was conjectured here to inhibit the ubiquitin-proteasome pathway and result in p53 accumulation in PCa cells. First, Western blotting was performed to detect total ubiquitination protein concentrations (Fig. 3a) and it was found that JS-K diminished the total ubiquitination protein as expected and, consistent with the present conjecture, p53 accumulation was observed (Fig. 3b). To discover whether JS-K accumulated p53 by inhibiting ubiquitin-proteasome degradation of p53 mediated by Mdm2, the Mdm2 protein concentration was first examined (Fig. 3b). Furthermore, p53 and Mdm2 interactions were also tested by co-immunoprecipitation (Co-IP, Fig. 3c). In addition, increased PARP cleavage in time-dependent manner was also detected (Fig. 3d), which could enhance p53 expression. Induction of caspase-9 and caspase-3 cleavage suggested that JS-K induced PCa cell apoptosis by influencing the mitochondrial apoptotic pathway. Therefore, JS-K’s effects on Bcl-2 family members were examined next and it was found that JS-K increased pro-apoptotic Bax protein concentrations and diminished anti-apoptotic protein Bcl-2 concentrations (Fig. 3d). All the quantitations of western blot results were presented in the Additional file 2: western blot results.

### JS-K inhibited transcription of specific AR target genes

**Fig. 4:**
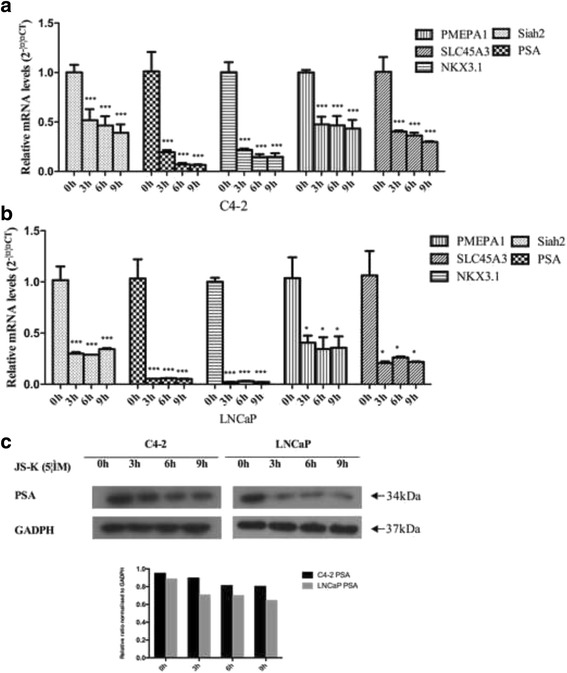
C4-2 (**a**) and LNCaP (**b**) cells were incubated for three periods (3, 6 and 9 h) with 5 μM JS-K. RT-PCR was performed to access the influence of JS-K on transcription of specific AR target genes (*PSA*, *NKX3.1*, *PMEPA1* and *SLC45A3*). Each assay was performed in triplicate and the expression levels of mRNAs were expressed as 2^-ΔΔCT^; **c** western blotting was performed to detect the influence of JS-K on PSA in C4-2 and LNCaP cells incubated for three periods (3, 6 and 9 h) with 5 μM JS-K. Results are mean ± SD of three different experiments. Single asterisks (*) indicate a significant difference (*P* < 0.05) and triple asterisks (***) indicates an extremely significant difference (*P* < 0.001)

Increased androgen receptor activity plays a major role in the progression of CRPC formation, which has always been thought to be a major obstacle to treatment of advance/metastatic prostate cancer. As previous experiments have shown that JS-K inhibited PCa cell proliferation, JS-K inhibition was predicted to involve AR activity prevention. To initially understand how JS-K inhibited transcripts of specific AR target genes, RT-PCR was performed to assess whether JS-K inhibited AR transcription activity. Transcripts of specific AR target genes (PMEPA1, PSA, SLC45A3, and NKX3.1) were clearly decreased (Fig. 4a and b), which suggested that JS-K showed potential inhibitory ability on AR transcriptional activity. Prostate specific antigen (PSA), the production and expression of which are highest in normal, benign hyperplastic, cancerous tissues of prostate, is well known as an AR transcriptional target. For further proof of JS-K transcriptional inhibition of specific AR target genes, Western blot analyses were performed to identify the PSA concentrations (Fig. 4c, Additional file 2: western blot results). These results revealed that JS-K inhibited PSA expression in a time-dependent manner.

### JS-K inhibited AR ubiquitination

**Fig. 5:**
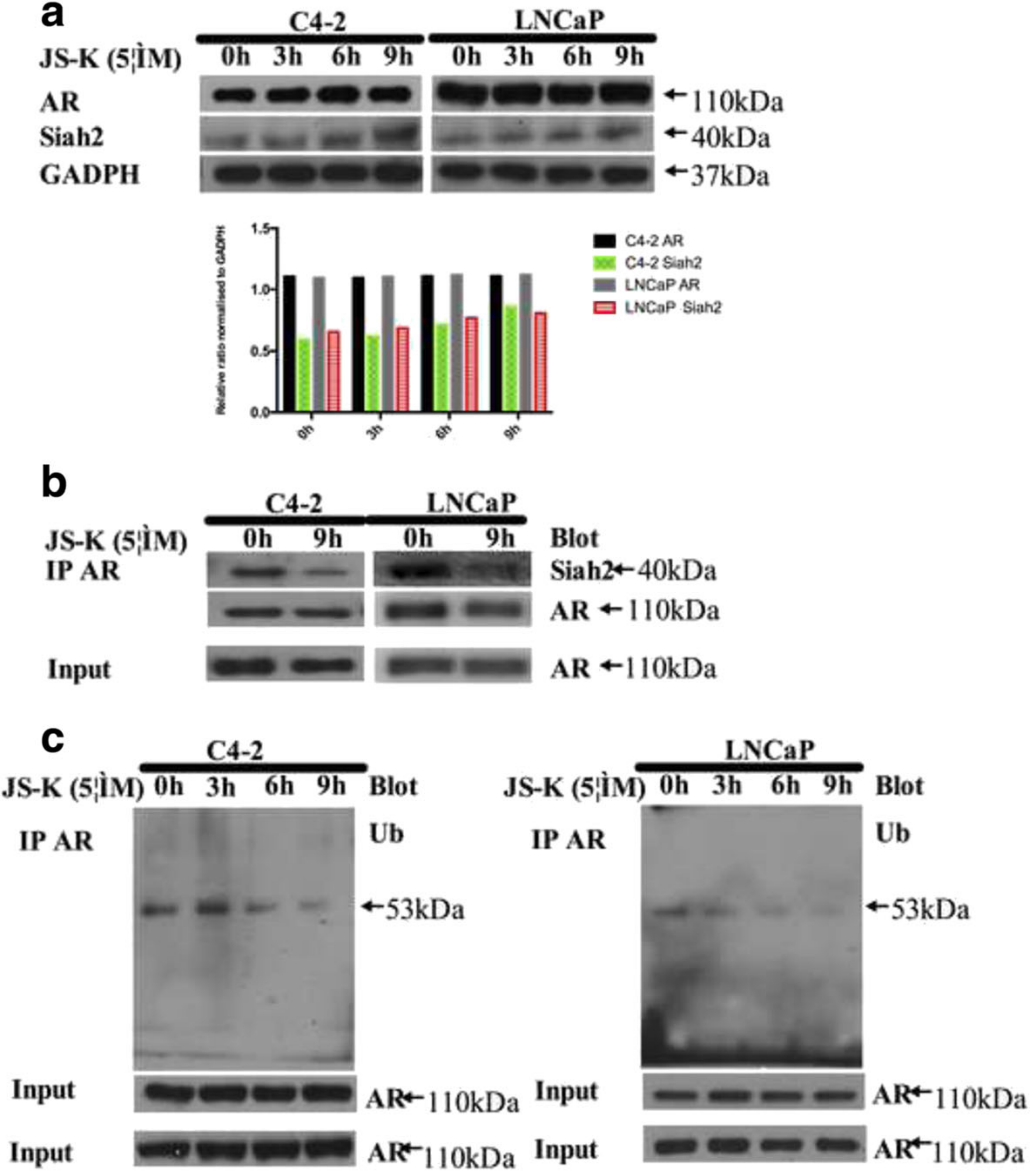
C4-2 and LNCaP cells were incubated for three periods (3, 6 and 9 h) with 5 μM JS-K; **a** Western blotting was performed to access the influence of JS-K on AR and Siah2 protein levels; **b** Co-IP was performed to access the influence of JS-K on combination between AR and Siah2 in C4-2 and LNCaP cells incubated for 9 h with 5 μM JS-K; **c** Co-IP was performed to access the influence of JS-K on combination of Ubiquitin and AR in C4-2 and LNCaP cells incubated for 9 h with 5 μM JS-K while maybe JS-K inhibited polyubiquitin of AR mediated by ubiquitin E3 ligase Siah2; All the IP were used in the input and at least three different experiments were performed

In humans, Siah2 regulates ubiquitination-dependent degradation of multiple substrates. Siah2-mediated proteasomal degradation of NCoR1-bound AR (transcriptionally inactive) on PSA promoter allows subsequent recruitment of p300-bound AR (transcriptionally active), leading to an increase in PSA gene transcription [14]. In addition, Siah2 auto-ubiquitylates itself and results in proteasomal degradation of Siah2 [24]. As it has been shown that JS-K inhibitsMdm2 and p53 interactions [21], JS-K was conjectured here to inhibit AR ubiquitination mediated by Siah2 and subsequently produced inhibition of ubiquitin proteasomal degradation of NCoR1-bound AR. Thus, Western blotting analyses were performed to identify Siah2 and AR concentrations and Co-IP performed to detect Siah2 and AR interactions. The resulting data indicated that JS-K increased Siah2 concentrations, which was similar to JS-K’s effects upon Mdm2, while AR concentrations did not change significantly in a time-dependent manner (Fig. 5a, Additional file 2: western blot results) and inhibited Siah2 and AR interactions (Fig. 5b). To further confirm this conjecture, ubiquitin and AR interactions were examined and it was found that JS-K significantly inhibited these interactions (Fig. 5c).

### JS-K stabilized NCoR1-bound AR and inhibitedp300-bound AR probably involved in regulating Siah2

**Fig. 6:**
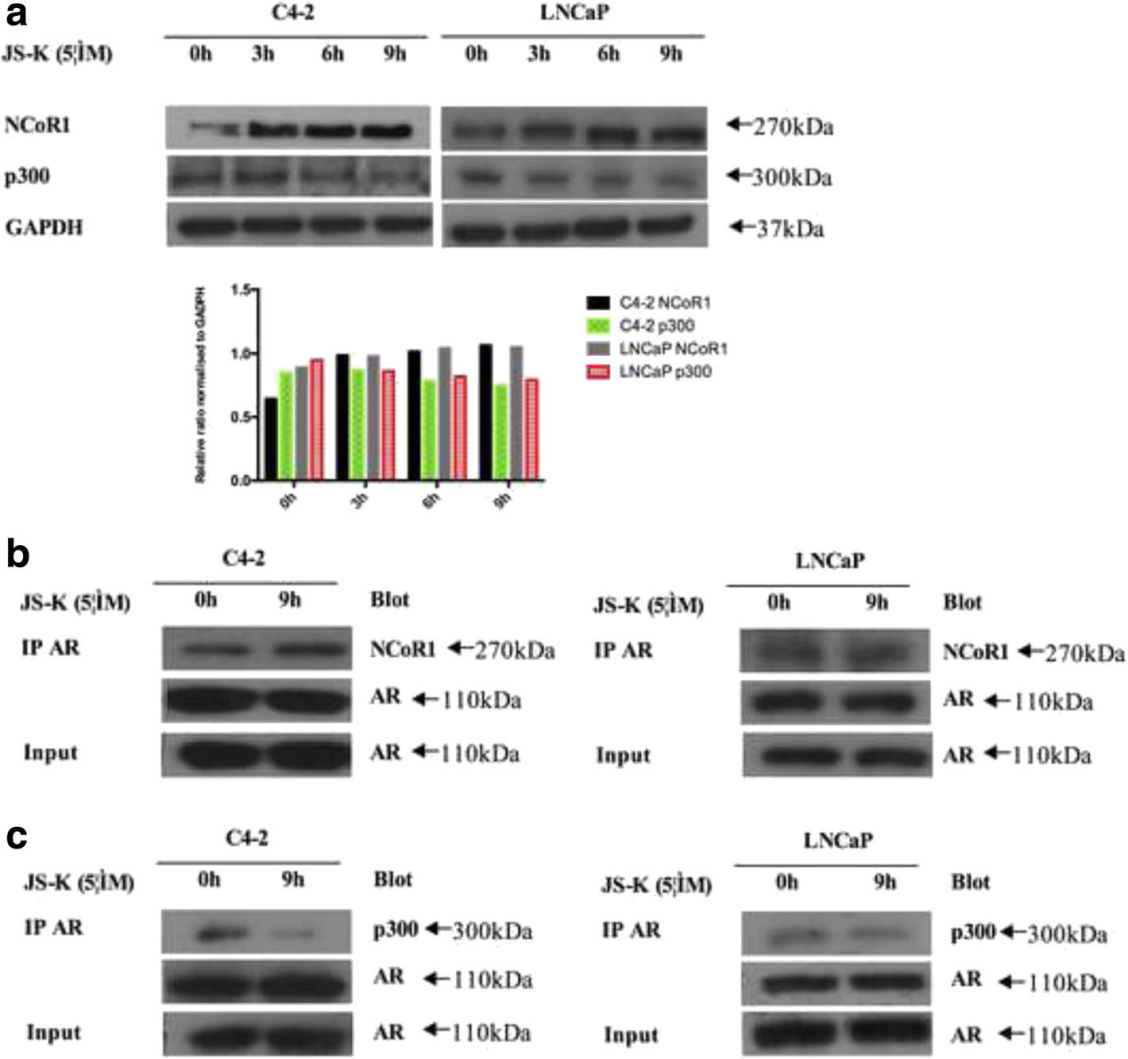
C4-2 and LNCaP cells were incubated for three periods (3, 6 and 9 h) with 5 μM JS-K; **a** Western blotting was performed to access the influence of JS-K on NCcR1 and p300 protein levels; **b** Co-IP was performed to access the influence of JS-K on combination between AR and NCoR1 in C4-2 and LNCaP cells incubated for 9 h with 5 μM JS-K; **c** Co-IP was performed to access the influence of JS-K on combination between AR and p300 in C4-2 and LNCaP cells incubated for 9 h with 5 μM JS-K; All the IP were used in the imput and at least three different experiments were performed

To understand if JS-K affected NCoR1 and p300 concentrations, Western blotting analyses were performed and it was found that NCoR1 concentrations increased while p300 concentrations diminished (Fig. 6a, Additional file 2: western blot results). To further understand Siah2 regulation of AR activity, the question of whether JS-K affected NCoR1 and AR interactions while also influencing AR and p300 interactions. Thus, Co-IP analyses were performed to detect JS-K’s influence on NCoR1-bound AR and p300-bound AR. Significantly, JS-K stabilized AR and NCoR1 interactions (Fig. 6b) and diminished AR and p300 interactions (Fig. 6c).

## Discussion

Recently, it has become known that current treatments of advanced PCa, based on androgen ablation therapies such as surgical and chemical castration, are very effective treatments initially, but almost all cases progress to CRPC eventually. Accumulating evidence has revealed that in nearly all cases resumption of AR transcription activity contributes to CRPC progression [25]. A recently identified mechanism, in which E3 ubiquitin ligase Siah2 regulates a subset of AR bound to corepressor NCoR1, results in removal of transcriptionally-inactive AR from chromatin and allows p300-bound AR binding to AREs, the mechanism of which has become the center of attention in PCa treatment investigations [14].

Interestingly, NO inhibition of AR-function in PCa cells was first described in vitro using the NO-donor DETA/NO. This study showed that NO inhibited AR-mediated genomic function by preventing its DNA-binding activity while not decreasing AR protein concentrations or decreasing nuclear AR translocation [26]. JS-K, activated by GST, which is frequently overexpressed in cancer tissue, is designed to release NO [8]. Accumulating investigations have revealed that JS-K affects apoptosis and proliferation in multiple types of cancer cells [9, 12, 27, 28], but JS-K’s mechanism for regulating PCa cells remains unclear. Therefore, the present study focused on JS-K’s possible effective mechanism upon PCa cell apoptosis and proliferation.

In this study, JS-K was shown to induce apoptosis in the PCa cell lines LNCaP and C4-2. As p53 operates as a key regulator in the apoptotic process, JS-K was reasonably expected to induce apoptosis by modulating p53. As is known, Mdm2, an ubiquitin ligase E3, is involved in p53 ubiquitin-proteasome degradation. In addition, evidence has shown that JS-K inhibits p53 degradation mediated by Mdm2 in RPE cells [21]. However, there have been no relevant reports that reveal JS-K’s impact on p53 ubiquitin-proteasome degradation mediated by Mdm2 in PCa cells. Therefore, here, JS-K was reasonably suspected to increase p53 concentrations by blocking the ubiquitin-proteasome pathway. Consistent with this conjecture, the present initial results revealed that JS-K increased p53 protein concentrations in PCa cell lines LNCaP and C4-2 in a time-dependent manner. Furthermore, JS-K regulation of p53 was verified as inhibiting the ubiquitin-proteasome degradation pathway in these cells by measurement of the total ubiquitin protein, and it was found to be diminished, which was consistent with the present conjecture. As increasing evidence has shown a clear association between Mdm2 and p53 [22, 29, 30], in the present study, p53 and Mdm2 interactions were also examined. In addition, to test whether JS-K activated the p53 mediated apoptosis pathway, Bcl-2 and Bax, which are involved the intrinsic mitochondrial apoptotic pathway, were examined. It was found that JS-K diminished anti-apoptotic protein Bcl-2 while increasing pro-apoptotic protein Bax, which led to activation of initiator caspase (usually caspase-9), which in turn activated executioner caspase-3 and initiated a caspase cascade reaction that eventually destroyed the cells.

A study has revealed that JS-K inhibits PCa cell proliferation through inhibition of the AR signaling pathway; this study is the only report reporting JS-K’s impact on PCa cells [10]. Cronauer et al. have revealed that NO inhibits AR-positive PCa cell proliferation significantly more effectively than AR-negative prostate cancer cell proliferation because NO inhibits AR DNA-binding activity [26]. In recent years, investigations of ubiquitin ligase E3 have highlighted them to be pivotal regulators of AR transcription activity in prostate cancer [14, 31–33]. For instance, ubiquitin E3 ligase RNF6 induces AR ubiquitination to increase AR transcriptional activity. In the meantime, Mdm2, SKP2, and CHIP, through ubiquitination and proteolysis, regulate AR. In recent years, Siah2 has been recognized as a regulator of AR transcriptional activity, with AR having been identified to be overexpressed in PCa cells. The results from the present study showed that JS-K inhibited the ubiquitin-proteasome degradation pathway in prostate cancer cells, resulting in reduction of total ubiquitin protein. Furthermore, Siah2 protein concentrations were examined to verify the supposition that JS-K inhibited Siah2 self-ubiquitin and accumulated protein concentrations just as JS-K affects Mdm2, as has been previously reported. In accordance with expectations, JS-K increased Siah2 concentrations, but it was found that JS-K exhibited clear proliferative inhibition of PCa cell lines LNCaP and C4-2. Thus, JS-K was suspected to diminish AR and Siah2 interactions while Siah2 was a pivotal proliferation regulator of AR. Co-IP results revealed that JS-K reduced AR and Siah2 interactions in these PCa cell lines. For further confirmation, Co-IP analyses to detect AR ubiquitination, which is regulated by Siah2, and it was found that JS-K reduced AR ubiquitination. As is known, Siah2 is a significant regulator involved in regulating ubiquitin-proteasome degradation of repressed AR-NCoR1 complexes while promoting active AR-p300 complex and AREs interactions. Therefore, AR and NCoR1 interactions were examined by Co-IP and the results showed that JS-K stabilized AR and NCoR1 interactions. These results supported the supposition that, here, JS-K might have inhibited Siah2’s ubiquitin ligase ability such that ubiquitin-proteasome degradation of AR-NCoR1 was blocked. In contrast to AR-NCoR1, AR-p300 complexes were further examined and it was found that JS-K decreased AR and p300 interactions. These results further supported the supposition that JS-K inhibited cell proliferation by regulating co-regulator and AR interactions, which subsequently targeted AREs and also then performed different functions.

## Conclusion

The present results suggested that JS-K was in a position to inhibit proliferation and induce apoptosis through probable regulation of the ubiquitin-proteasome degradation pathway. Taken together, it would be of high interest to further investigate whether JS-K regulates interactions between AR-bound co-regulators and AREs of specific AR target genes. These findings warrant further investigation to facilitate potential development of AR-based prognostic and therapeutic approaches.

## Additional files

Additional file 1: IC50 results. The IC_50_ of LNCaP and C4-2 cell lines that treated by JS-K. (XLSX 10 kb)

Additional file 2: western blot results. The quantitation of western blot of relative protein of LNCaP and C4-2 cell lines that treated by JS-K. (XLSX 38 kb)

## Abbreviations

AR: Androgen receptor
AREs: Androgen response elements
ARGs: AR responsive genes
CRPC: Castration-resistant prostate cancer
E1: Ubiquitin-activating enzyme
E2: Ubiquitin-conjugating enzyme
E3: Ubiquitin-protein ligase
GST: Glutathione-S-transferase
NO: Nitric oxide
PCa: Prostate cancer
PSA: Prostate specific antigen
